# Transactions of the New York Odontological Society

**Published:** 1875-05

**Authors:** 


### BIBLIOGRAPHICAL.

*Transactions of the New York Odontological Society.*—Special Meeting, December 14th, 15th and 16th, 1874. Published in a very neat and attractive style by Samuel S. White, Philadelphia, 1875.

The officers of this Society are: President—A. L. Northrop; Vice-President—Benjamin Lord; Recording Secretary—Wm. Jarvis, Jr; Corresponding Secretary—Wm. Carr; Executive Committee—W. A. Bronson, E. A. Bogue, C. E. Francis.

This work contains a number of interesting and instructive essays and is worthy of careful perusal.

